# A Paper-Based Electrochromic Array for Visualized Electrochemical Sensing

**DOI:** 10.3390/s17020276

**Published:** 2017-01-31

**Authors:** Fengling Zhang, Tianyi Cai, Liang Ma, Liyuan Zhan, Hong Liu

**Affiliations:** 1 State Key Laboratory of Bioelectronics, School of Biological Science and Medical Engineering, Southeast University, Nanjing 210096, China; 220143702@seu.edu.cn (F.Z.); 213143120@seu.edu.cn (T.C.); 213140698@seu.edu.cn (L.M.); 213143432@seu.edu.cn (L.Z.); 2 Laboratory of Environment and Biosafety, Research Institute of Southeast University in Suzhou, Suzhou 215123, China

**Keywords:** electrochromism, paper, battery, POCT

## Abstract

We report a battery-powered, paper-based electrochromic array for visualized electrochemical sensing. The paper-based sensing system consists of six parallel electrochemical cells, which are powered by an aluminum-air battery. Each single electrochemical cell uses a Prussian Blue spot electrodeposited on an indium-doped tin oxide thin film as the electrochromic indicator. Each electrochemical cell is preloaded with increasing amounts of analyte. The sample activates the battery for the sensing. Both the preloaded analyte and the analyte in the sample initiate the color change of Prussian Blue to Prussian White. With a reaction time of 60 s, the number of electrochemical cells with complete color changes is correlated to the concentration of analyte in the sample. As a proof-of-concept analyte, lactic acid was detected semi-quantitatively using the naked eye.

## 1. Introduction

With the improvement of people’s living standards and the growing world-wide problem of an aging population, people’s demand for health care and the lack of medical resources have driven the development of point-of-care testing (POCT). POCT refers to on-site tests that are provided by non-professional personnel outside a central laboratory. It simplifies the test procedure and replaces sophisticated instruments with affordable, portable and user-friendly devices [1]. Therefore, POCT has been widely used for biomedical diagnosis under resource-limited conditions [2]. For example, glucometers, which are electrochemical biosensors, are widely used for self-monitoring of blood glucose level by diabetes patients [3,4]. Lateral-flow immunochoramatographic test strips have been used for detection of a varieties of biomarkers such as human chorionic gonadotropin for pregnancy tests [5,6].

As a promising solution to the need for POCT under resource-limited conditions, paper-based microfluidic sensors have attracted considerable research interest [7,8,9], and have now contributed to a number of interesting applications in diagnostics, environmental monitoring, and food safety [10,11]. Conventional paper-based microfluidic sensors are based on visual (colorimetric) or fluorescent readouts. However, the visual detection usually provides only qualitative results, and the detection of fluorescence signal relies on sophisticated instruments, which drives the development of paper-based electrochemical microfluidic sensors [12,13]. Although electrochemical detection is inherently quantitative, the design principle for paper-based electrochemical microfluidic sensors that have been reported so far involves a disposable paper fluidic system that is controlled and readout using a conventional potentiostat [12,13] or commercial reader [14,15]. The requirement for instruments makes them more expensive and less compatible with the POCT concept, especially in underdeveloped countries or remote areas where power supply facilities are not well established.

To address these issues, we have previously reported a method for fully integrating paper-based electrochemical microfluidic devices with an on-board metal/air battery and an electrochromic electrode for transducing electrochemical signals to visual readouts [16]. However, like colorimetric detection, this device only provided qualitative test results. For diagnostics, quantitative or at least semi-quantitative detection is preferred for some biochemical indicators (e.g., lactic acid (LA), glucose and uric acid), because an abnormal elevation or decline of the level indicates the occurrence of disease(s).

**Figure 1:**
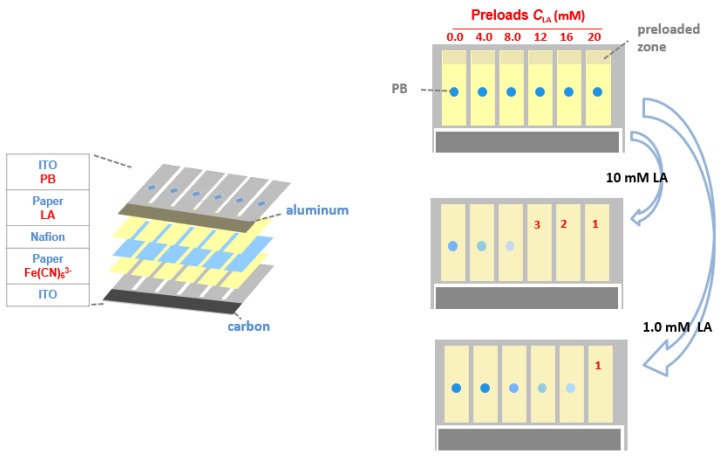
Schematic illustration showing the structure and working principle of the proposed electrochromic sensing array. It consists of two parts: an electrochemical sensor and an aluminum-air battery for powering the sensor. The sensor is composed of two ITO films as electrodes, two pieces of paper as the detection reservoirs and a Nafion film as the ionic conductor. One of the ITO electrodes has electrodeposited PB on it as the electrochromic indicator. The paper reservoir contacted with the PB is preloaded with increasing amounts of standard LA solution (0.0, 4.0, 8.0, 12, 16, 20 mM). The other is preloaded with dried LOx and Fe(CN)_6_^3−^. The catalytic oxidation of LA by LOx results in conversion of Fe(CN)_6_^3−^ to Fe(CN)_6_^4−^. Fe(CN)_6_^4−^ is then oxidized back to Fe(CN)_6_^3−^ on the ITO electrode, resulting in conversion of PB to colorless PW on the ITO electrode. When the concentration of LA exceeds a threshold value, PB can be converted to PW completely. Higher LA concentration leads to more spots that changes color, which enables semiquantitative detection of LA using the naked eye.

Herein we introduce a paper-based electrochromic array with an inexpensive, on-board battery for visualized electrochemical sensing. The operational principles of the electrochromic array are illustrated in Figure 1. The array consists of six parallel electrochemical cells, which are powered by an aluminum-air battery. Each single electrochemical cell uses a Prussian Blue (PB) spot electrodeposited on an indium-doped tin oxide (ITO) thin film as the electrochromic indicator. The chromatographic papers of each cell not only form electrical contact via a pair of transparent ITO electrodes, but also act as reaction reservoirs. The paper is preloaded with dried lactate oxidase (LOx), Fe(CN)_6_^3−^ and increasing amounts of LA. The array is activated by introduction of an LA sample, which acts both as the matrix for the analyte and the electrolyte for the battery. After sample introduction, LOx catalyzes the oxidation of the LA by the Fe(CN)_6_^3−^ which leads to the production of Fe(CN)_6_^4−^. Under the potential provided by the aluminum−air battery, PB is reduced to colorless Prussian White (PW) at the cathode, with simultaneous oxidization of Fe(CN)_6_^4^^−^ at the anode. Because both the preloaded LA and the LA in the sample initiate the color change of PB to PW, with a reaction time of 60 s, the number of electrochemical cells with complete color changes can be correlated to the concentration of analyte in the sample.

The electrochromic sensing array we report here gives semiquantitative test results using the naked eye with no additional detection device or instruction being required. The electrochromic sensing array is simple, low-cost and self-powered, requiring no external power source. The underlying detection principle can be applied to detection of a variety of analytes (e.g., glucose, uric acid) with the use of corresponding oxidase. Therefore, we believe the paper-based electrochromic sensing array represents a promising solution to POCT under resource-limited conditions.

## 2. Experimental Section

### 2.1. Chemicals and Materials 

K_3_Fe(CN)_6_, FeCl_3_·6H_2_O, HCl, L-(+)-lactic acid, lactate oxidase from Pediococcus and concentrated phosphate buffered saline (PBS, pH 7.4) were purchased from Sigma-Aldrich (Shanghai, China). ITO/PET thin film electrodes were purchased from Zhuhai Kaivo Optoelectronic Technology Co., Ltd. (Guangzhou, China). A laser cutting machine from Huatai Laser Engraving Co., Ltd. (Shenzhen, China) was used for device fabrication. Conductive carbon ink (Creative Materials Inc., Tyngsboro, MA, USA) was used to fabricate the battery cathode. Aluminum foils (Reynolds Wrap, Fisher Scientific, Shenzhen, China) were used to fabricate the battery anode. Double-faced adhesive tape was purchased from Xinyidai Viscose Technology Co., Ltd. (Shenzhen, China). Chromatographic paper (grade 1) was obtained from Whatman (Shanghai, China). A Nafion membrane (N-115, Fuel Cell Store, Inc., Boulder, CO, USA) was used to separate the anodic and cathodic reactions in the sensing reservoir while maintaining ionic conductivity. All solutions were prepared with redistilled water. All reagents were used as received without further purification.

### 2.2. Experimental Procedures

The electrochromic sensing system was composed of an aluminum-air battery and an electrochromic sensing array (Figure 1). The battery cathode was fabricated using activated carbon ink with high surface area. The carbon material was coated onto the specific zone (2.0 mm × 20 mm) of an ITO electrode via blade coating, and then cured at 40 °C on a hotplate (Shanghai Sile Instrument Co., Ltd., Shanghai, China) for 30 min. The battery anode was fabricated by attaching a piece of aluminum foil (2.0 mm × 20 mm) onto an ITO electrode. A chromatographic paper was used to separate the anode and cathode and provided reservoir to store electrolyte solution. The open circuit voltage (OCV) of the battery was measured using a CHI 900D potentiostat (CH Instruments, Shanghai, China).

The electrochromic array (10 mm × 20 mm) had five layers. The two-electrode electrochemical cell was constructed using one ITO electrode that was modified with PB as the electrochromic indicator and another ITO electrode without surface modification. A piece of chromatographic paper was sandwiched between these two ITO electrodes, which defined the sensing and battery reservoirs. A Nafion film was used to separate the sensing reagents from the ITO electrode on which the PB spots were immobilized. Double faced adhesive tape was used to clamp the ITO electrodes so that all layers were in contact with one-another. 18 μL aliquot of PBS solution (pH 7.4) containing 500 mM K_3_Fe(CN)_6_ and 250 U/mL LOx was dropcast onto the paper reservoirs on the side of the naked ITO electrode and then dried at 20 °C. Then 3.0 μL aliquots of solution containing of LA at known concentrations were preloaded on each electrochromic cell. From left to right (Figure 1), the preloaded LA concentration was 0.0, 4.0, 8.0, 12, 16, 20 mM, respectively. Electrodeposition of PB on an ITO electrode was carried out as follows: first, a double-faced adhesive tape mask having holes with a diameter of 1.12 mm was patterned using the laser cutter. Second, electrodeposition of PB onto the ITO through the holes was carried out using a previously reported procedure [17]. Briefly, a plating solution containing 2.6 mM HCl, 10 mM K_3_Fe(CN)_6_, and 10 mM FeCl_3_ was freshly prepared, and then a 40 μL aliquot of the plating solution was dropcast onto the holes of the mask. Electrodeposition was carried out for 60 s at a constant cathodic current of 1.0 μA/mm^2^ using a CHI 900D potentiostat and a Pt electrode as the reference and counter electrode. After deposition, the mask was removed, the ITO electrode was washed with deionized water, and then it was dried under nitrogen gas.

The electrochemical sensing was initiated after introduction of the LA sample into the sensing reservoirs. Upon injection of battery electrolyte, the battery was then activated to report the sensing result. To measure the color intensity of PB spot, we used ImageJ software. Firstly, the original photograph of the spot was imported into the software and was turned into grey-scale format. After color reversal, the PB spot was selected, and the average intensity of each PB spot was shown in the software.

## 3. Results and Discussion

Conventional colorimetric detection of paper-based microfluidics is simple and convenient, but the quantification of colorimetric test results is complicated and is usually biased due to background light. Electrochemical detection is intrinsically quantitative because current replaces the color change as the readout signal. However, in most cases, electrochemical paper-based microfluidics sensors still need a conventional potentiostat [12,13] or commercial reader [14,15] to read the result, which make it less compatible with POCT under resource-limited conditions. In this work, we developed an electrochromic sensing array to achieve semi-quantitative electrochemical detection while maintain the simplicity of naked-eye detection.

**Figure 2:**
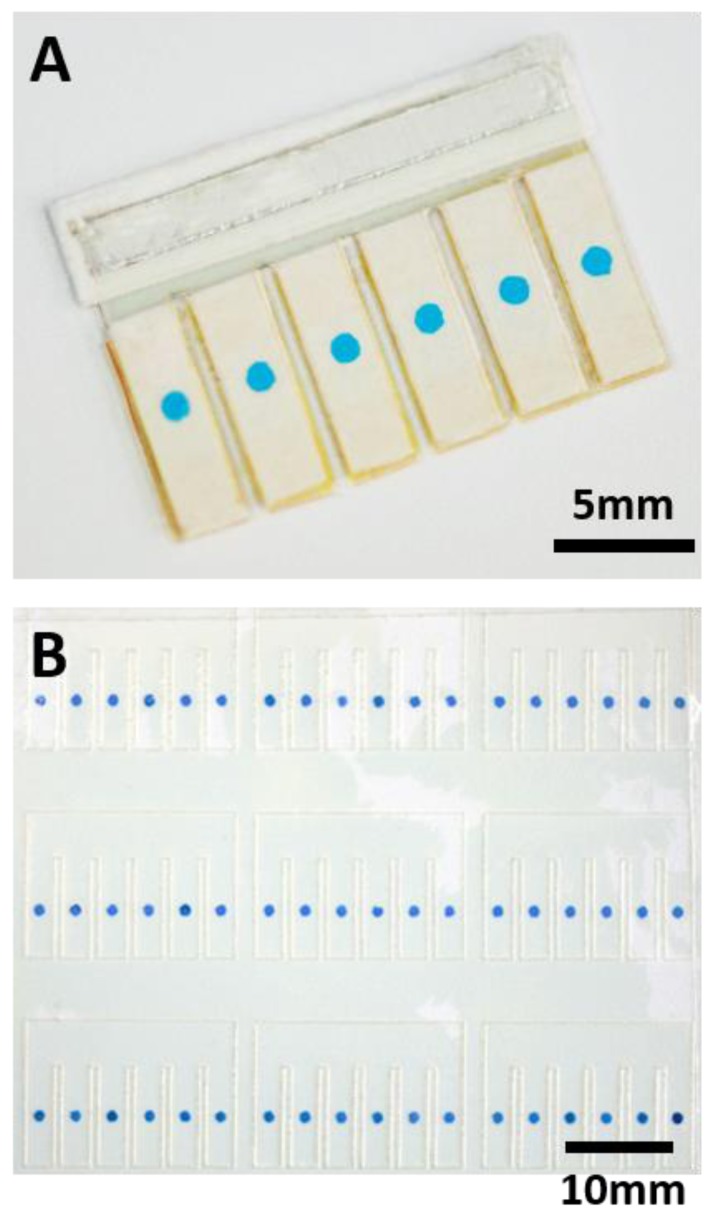
(**A**) Photograph of the electrochromic sensing array for LA detection; (**B**) Photograph of the PB spots simultaneously electrodeposited on an ITO film substrate. The electrochromic array contains 54 PB spots, which can be further cut and used to fabricate 9 sensing devices.

As shown in Figure 1, the electrochromic sensing array was composed of six electrolytic cells. Each cell was powered by the aluminum battery integrated on the chip. Each sensor was composed of two ITO electrodes. The resistivity of each ITO electrode is about 35 Ω/sq, measured by the electrode supplier. Between the two ITO electrodes were paper layers with preloaded reagents and a Nafion layer for maintaining ionic conductivity and separating the reagents from unwanted mixing. On the sensing array, 10 μL LA sample was introduced into the paper reservoir of the battery by capillary force to activate the battery. Since the battery shared the same ITO electrodes with the electrochromic sensing array, the battery provided the electrical energy for the electrolytic reactions of the sensing array. Then 18 μL LA sample was introduced into the paper layers of the sensing array so that the preloaded reagents (LOx and Fe(CN)_6_^3−^) on one of two ITO electrodes (anode) reacted with LA in the sample that resulted in the production of Fe(CN)_6_^4−^. The other ITO electrode (cathode) was modified with a PB spots by electrodeposition. The PB which changed to colorless upon reduction was used as an electrochromic indicator for reporting the occurrence of oxidation event (Fe(CN)_6_^4−^ oxidation) on the anode. Since increasing amounts of LA were preloaded onto the paper layers of the sensing array, the number of PB spots that changed color at a given time was determined by the concentration of LA in the sample. Higher LA concentration leads to more spots changing color, which enables semiquantitative detection of LA using the naked eye. Figure 2A shows a real sensor for LA detection.

PB has been widely used in electrochemical sensing systems for modifying electrodes owing to its electrochromic behavior, stability, electrochemical reversibility and biocompatibility [18,19]. The relationship between the charge stored in the PB spot and detection concentration of analyte was reported previously (Equation (1)) [16]:

(1) $$\frac{a\pi d^{2}Q}{4}=\;nFVC$$

where, *Q* is the charge density required for color transition of PB in C/mm^2^, *d* is the diameter of PB spot in mm, *n* is the number of electrons transferred in reaction, *F* is the Faraday constant, *V* is the volume of sample in L, and a is a dimensionless empirical constant related to experimental conditions such as sensing time and mass transfer.

$$\frac{a\pi d^{2}Q}{4}=\;nFVC$$

In LA detection, *n* = 2. Since the normal range of LA in sweat is from 0 to 20 mM, the maximum concentration of LA is 20 mM. The volume of LA sample is 3.0 μL. The diameter of PB spot in each sensor, *d*, is 1.12 mm. For the devices reported here, *Q* is given by Equation (2):

(2) $$Q=\frac{5.88\times 10^{-3}C}{a}=\frac{0.1176}{a}$$

In our experiments, the sensing time is 60 s. By optimizing the experimental conditions, we determined that the charge density *Q* of PB is 61 µC/mm^2^ (current density of 1.0 µA/mm^2^ for 60 s) when PB is converted to its colorless form with 20 mM LA in the sample. The total charge used to deposit the PB spots for each device reported here was uniform to ensure reproducible color changes. Note that the amount of charge stored in the PB spot can be adjusted to match the detection limit and range of the sensor for specific clinical needs.

To demonstrate the electrochromic array can be easily prepared, we attempted to electrodeposit large amount of PB spots in parallel. We patterned large numbers of ITO electrodes in an ITO film substrate using a laser cutter, and made sure that the electrodes did not drop off from the substrate. Next, we patterned the double-faced adhesive tape mask using laser cutter and aligned with the ITO electrodes. We fabricated a rectangular reservoir for electrodeposition of PB, and put the ITO film into the reservoir. Finally, the reaction reservoir was filled with plating solution, and electrodeposition was carried out using another ITO film as reference electrode. As shown in Figure 2B, an electrochromic array containing 54 PB spots were fabricated simultaneously. They can be further cut and used to fabricate nine sensing devices. To evaluate the reproducibility of this method, we analyzed the intensity of each PB spot using ImageJ software. The average intensity of PB spots was 110, and standard deviation was 10, so the relative error is about 9%, which is acceptable.

**Figure 3:**
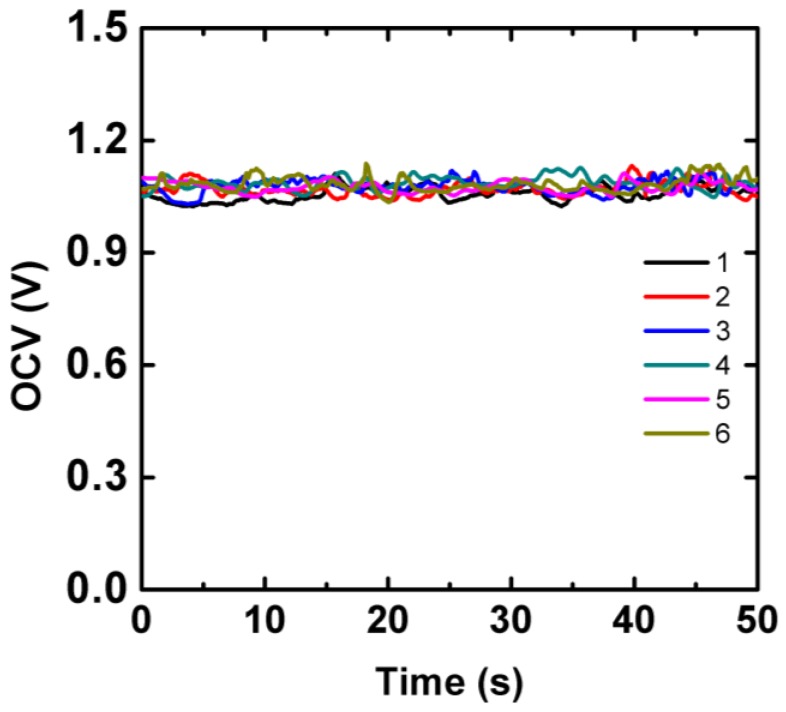
The average OCV of the aluminum-air battery as a function of time measured from six individual cells.

Power supply is a critical concern for electrochemical POCT under resource-limited conditions, which have driven the development of inexpensive, disposable paper-based batteries [14,15,16]. Here we integrated aluminum-air battery on the chip to provide electrical energy for the electrochromic sensing array. The aluminum foil is easy to obtain and the output voltage was about 1.0 V, which just meets requirement of the sensor. The aluminum-air battery was composed of a piece of aluminum foil attached onto the ITO electrode as the anode. The cathode was ITO electrode modified with activated carbon that had large surface area for oxygen reduction. A layer of chromatographic paper was sandwiched between the ITO electrodes as electrolyte reservoir. The battery was activated by introduction of 10 µL aliquot of LA sample into the battery paper reservoir. As shown in Figure 3, the average OCV of the aluminum-air battery measured from six individual cells was about 1.07 V with a maximum peak-to-peak variation of 70 mV during 50 s.

**Figure 4:**
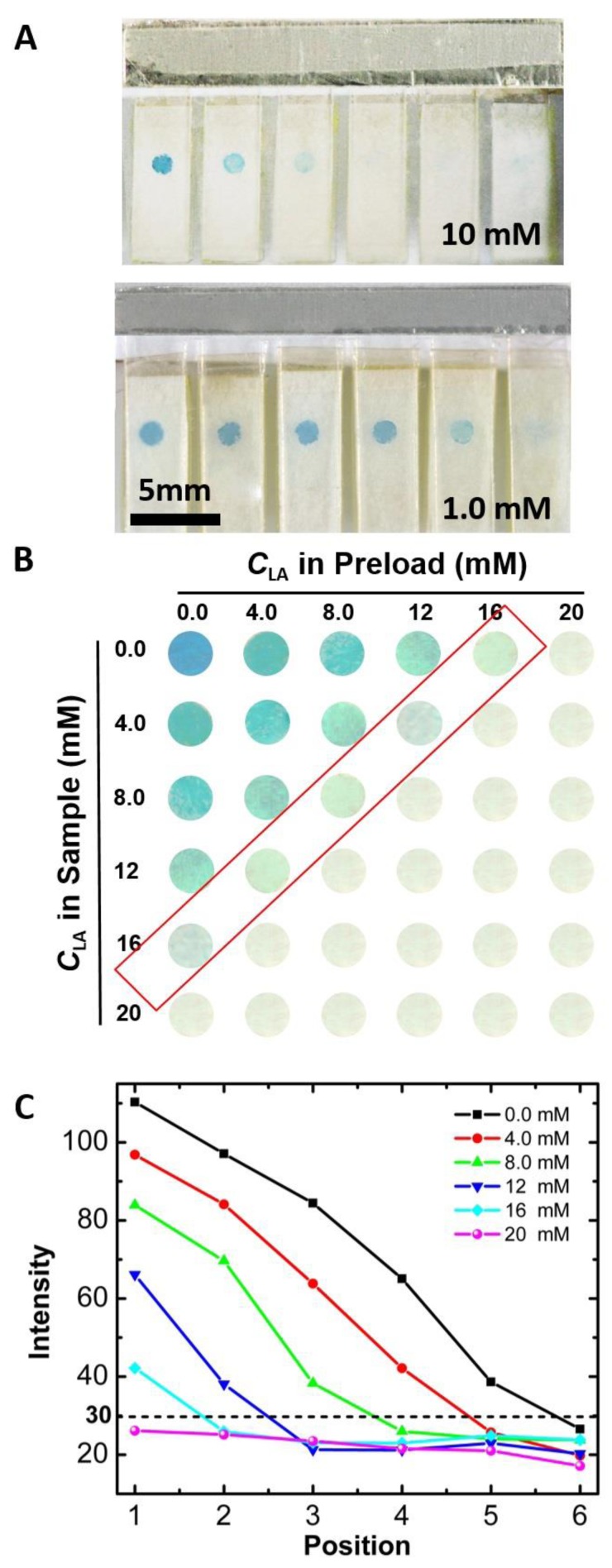
(**A**) Photographs of LA electrochromic sensing array after introduction of 18 μL aliquot of sample and allowing to react for 60 s. The concentration of LA in the sample is indicated. The number of PB spots which turned from blue to colorless increased due to the increase of LA concentration; (**B**) Photographs of PB spots on the LA electrochromic array after introduction of 18 μL aliquot of sample containing LA at different concentrations from 0.0 mM to 20 mM, respectively; (**C**) The color intensity of the PB spots shown in (B) as a function of LA concentration.

Figure 4A displays the testing results of the electrochromic array after introduction of 18 µL samples containing LA at 10 mM and 1.0 mM, respectively. As we have discussed earlier, the PB spot can be completely converted to PW when the total concentration of LA (preloaded LA plus LA in the sample) was more than 20 mM. When the concentration of LA in the sample was 10 mM, the total concentrations of LA in the sensing array were 10, 14, 18, 22, 26, and 30 mM, respectively. Therefore, the number of cells that underwent color change was three (Figure 4A, top row). Similarly, when the LA concentration in sample was 1.0 mM, the number of cells that underwent the color change was one (Figure 4A, bottom row). Using the sensing device, different samples were tested and the number of cells that underwent color change was correlated to LA concentration in the sample. In Figure 4B, each row shows the testing result of a sample. From top to bottom, the LA concentrations in the sample were 0.0, 4.0, 8.0, 12, 16, 20 mM, respectively. The testing results can be directly interpreted by the naked eye for semiquantitative detection of LA.

To completely eliminate any subjectivity from the result analysis, the color intensity of each spot can be analyzed using ImageJ software. As shown in Figure 4C, the color intensity of each spot is plotted and correlated to the concentration of LA in the sample. With higher LA concentrations, the color intensity of more PB spots fell below 30, which is the threshold for determining whether the PB spot completely changes from blue to colorless. Note that the detection range can be adjusted by three ways: (1) changing the amount of LA preloaded; (2) changing the amount of charge stored in the PB spots; (3) changing the number of electrodes in the array. Therefore, the electrochromic sensing array we reported here provides a versatile platform for semiquantitative electrochemical sensing.

**Figure 5:**
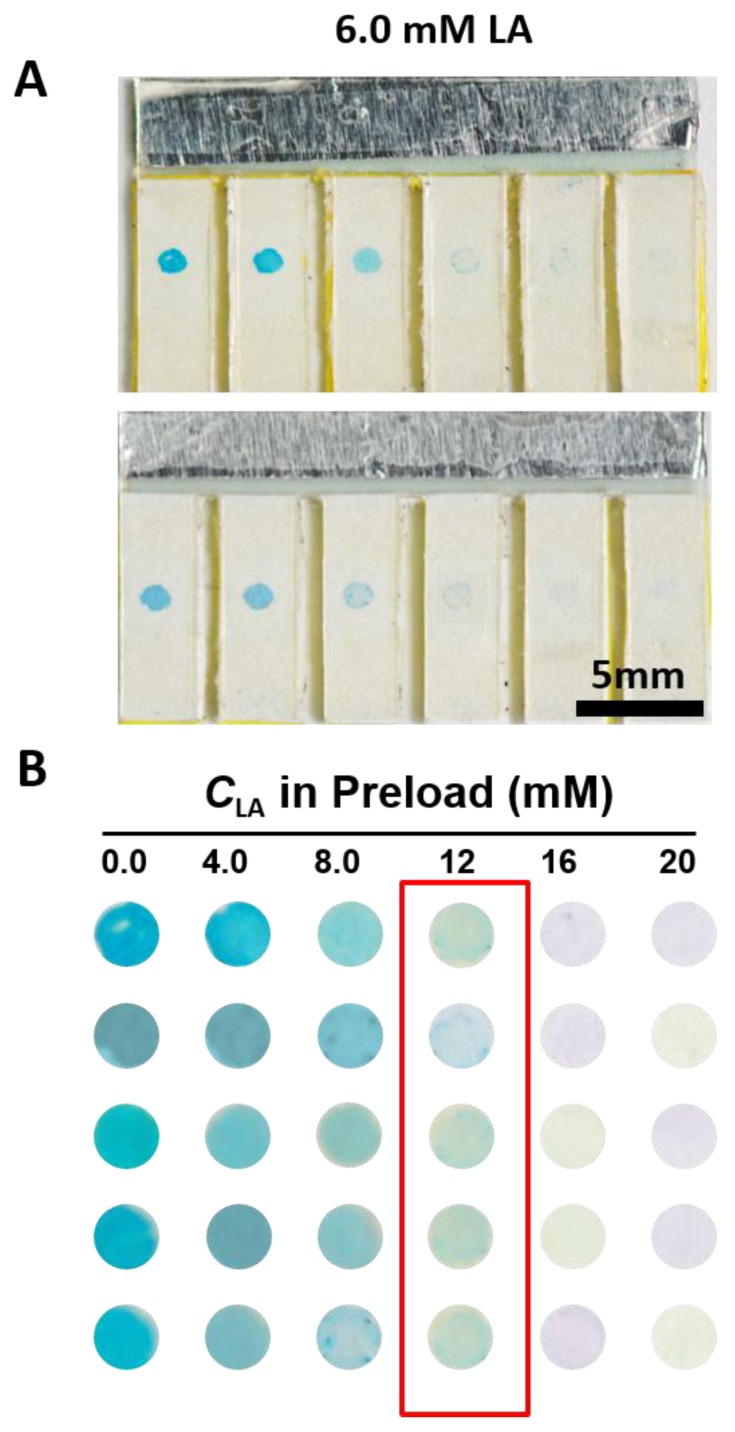
(**A**) Photographs of the LA electrochromic sensing device for two replicated tests of a sample containing LA at 6.0 mM; (**B**) Photographs of the electrochromic PB spots for replicated tests of a sample containing LA at 6.0 mM.

To test the reproducibility of the electrochromic array, we carried out the repeated detection of a sample containing LA at 6.0 mM. As shown in Figure 5, even though there is slight variation in the color intensities of the PB spots, the number of cells that underwent complete color change was always two in all of the replicate tests, so the reproducibility of the sensor is acceptable.

## 4. Summary and Conclusions

We have reported a paper-based electrochromic sensing array for visualized semi-quantitative detection. The paper-based sensing system consists of parallel electrochemical cells which are powered by an aluminum-air battery. Each electrochemical cell is preloaded with increasing amounts of analyte. Both the preloaded analyte and the analyte in the sample initiate the color change of Prussian Blue to Prussian White on the ITO indicating electrode. After completion of the assay, the number of electrochemical cells with complete color changes can be correlated to the concentration of analyte in the sample. As a proof-of-concept analyte, LA is detected semi-quantitatively using the naked eye. The sensor is inexpensive, self-powered and can be assembled on a lab bench without sophisticated instruments, which provides an inexpensive and versatile platform for electrochemical POCT under resource-limited conditions.
